# The Homogeneous Multiplexed System-a New Method for Autoantibody Profile in Systemic Lupus Erythematosus

**DOI:** 10.1080/17402520500116723

**Published:** 2005-06

**Authors:** Gisele Zandman-Goddard, Boris Gilburd, Ora Shovman, Miri Blank, Svetlana Berdichevski, Pnina Langevitz, Yehuda Shoenfeld

**Affiliations:** 1 The Center for Autoimmune Diseases and the Department of Medicine B Sheba Medical Center Sackler Faculty of Medicine Tel-Aviv University Israel; 2 Department of Rheumatology Unit Sheba Medical Center Sackler Faculty of Medicine Tel-Aviv University Israel

## Abstract

Systemic lupus erythematosus (SLE) is a multi-systemic autoimmune disease leading to immunological aberrations and excessive multiple autoantibody production. The aim of this study was to investigate the prevalence of multiple autoantibodies in SLE patients utilizing the multiplex system method.

We analyzed the presence of elevated titers of anti-Ro, anti-La, anti-RNP, anti-Sm, anti-Jo1, anti-centromere, anti-Scl-70, anti-histone, and anti-dsDNA antibodies in 199 serum samples (113 SLE patients, 86 healthy donors). We compared the type, level and number of autoantibodies and the correlation between the autoantibody profile and disease severity utilizing the SLEDAI score.

Elevated titers of at least one autoantibody were detected in 48% of 42 SLE patients. Elevated titers of anti-Ro antibodies were most commonly detected. The distribution of specific autoantibodies was: anti-Ro- 23.8%, anti-dsDNA- 19%, anti-histone- 19%, anti-RNP- 14.2%, anti-La antibodies- 11.9%, anti-Sm- 7.1%, anti-Scl 70-4.7%, and anti-centromere- 2.4%. Utilizing ROC analysis, the sensitivity and specificity of anti-DNA antibodies at a cutoff value of 34 IU/ml were 87.1% and 79.4% respectively. Elevated titers of anti-Jo1 antibody were not detected. There was a correlation with the titer of anti-Ro antibodies and disease activity by the SLEDAI score. Seven patients harbored one autoantibody only, 15 patients harbored 2-3 autoantibodies, 3 patients harbored 4-5 autoantibodies, and one patient harbored 6 autoantibodies. A correlation between the number of autoantibodies per patient and disease severity was found. One patient with a multitude of autoantibodies had severe lupus and a myriad of clinical manifestations.

In conclusion, the multiplex system is specific and sensitive, provides an autoantibody profile in a single test, and may be useful as a diagnostic test for SLE. Elevated anti-Ro antibodies are associated with severe disease. An autoantibody load may be indicative of more severe disease.


*Clinical & Developmental Immunology*, June 2005; 12(2): 107–111

Taylor & Francis

Taylor & Francis Group

### The homogeneous multiplexed system–a new method for autoantibody profile in systemic lupus erythematosus

GISELE ZANDMAN-GODDARD[1], BORIS GILBURD[1], ORA SHOVMAN[1], MIRI BLANK[1], SVETLANA BERDICHEVSKI[1], PNINA LANGEVITZ[2], & YEHUDA SHOENFELD[1,†]

[1]*The Center for Autoimmune Diseases and the Department of Medicine B, Sheba Medical Center; Sackler Faculty of Medicine, Tel-Aviv University, Israel, and* [2]*Department of Rheumatology Unit, Sheba Medical Center; Sackler Faculty of Medicine, Tel-Aviv University, Israel*

**Abstract**

Systemic lupus erythematosus (SLE) is a multi-systemic autoimmune disease leading to immunological aberrations and excessive multiple autoantibody production. The aim of this study was to investigate the prevalence of multiple autoantibodies in SLE patients utilizing the multiplex system method.

We analyzed the presence of elevated titers of anti-Ro, anti-La, anti-RNP, anti-Sm, anti-Jo1, anti-centromere, anti-Scl-70, anti-histone, and anti-dsDNA antibodies in 199 serum samples (113 SLE patients, 86 healthy donors). We compared the type, level and number of autoantibodies and the correlation between the autoantibody profile and disease severity utilizing the SLEDAI score.

Elevated titers of at least one autoantibody were detected in 48% of 42 SLE patients. Elevated titers of anti-Ro antibodies were most commonly detected. The distribution of specific autoantibodies was: anti-Ro- 23.8%, anti-dsDNA- 19%, anti-histone- 19%, anti-RNP- 14.2%, anti-La antibodies- 11.9%, anti-Sm- 7.1%, anti-Scl 70-4.7%, and anti-centromere- 2.4%. Utilizing ROC analysis, the sensitivity and specificity of anti-DNA antibodies at a cutoff value of 34 IU/ml were 87.1% and 79.4% respectively. Elevated titers of anti-Jo1 antibody were not detected. There was a correlation with the titer of anti-Ro antibodies and disease activity by the SLEDAI score. Seven patients harbored one autoantibody only, 15 patients harbored 2–3 autoantibodies, 3 patients harbored 4–5 autoantibodies, and one patient harbored 6 autoantibodies. A correlation between the number of autoantibodies per patient and disease severity was found. One patient with a multitude of autoantibodies had severe lupus and a myriad of clinical manifestations.

In conclusion, the multiplex system is specific and sensitive, provides an autoantibody profile in a single test, and may be useful as a diagnostic test for SLE. Elevated anti-Ro antibodies are associated with severe disease. An autoantibody load may be indicative of more severe disease.

**Keywords:** *Systemic lupus erythematosus, multiplex bead array, autoantibody profile, AtheNA Multi-Lyte ANA system*

### Introduction

Systemic lupus erythematosus (SLE) is a multi-system autoimmune disease with diverse clinical and laboratory features. SLE patients may have mild disease characterized by joint and cutaneous involvement, moderate disease when there is serositis or hematological involvement (thrombocytopenia, anemia, leukopenia) or severe disease manifested by injury to the renal tissue or central nervous system. SLE is classically characterized by elevated titers of antinuclear antibodies (ANA) and anti-dsDNA antibodies. The autoantibody profile may include a response to other extractable nuclear antigens (ENA) including Ro/SSA, La/SSB, RNP, and Sm. The autoantibody profile is usually established by the ELISA method where the patient's serum is tested for

Correspondence: Y. Shoenfeld, Department of Medicine B, Sheba Medical Center, Tel Hashomer 52621, Israel. Tel: 972 3 5302652. Fax: 972 3 5352855. E-mail: shoenfel@post.tau.ac.il

†Incumbent of the Laura Schwarz-Kipp Chair for Research in Autoimmunity, Tel-Aviv University, Israel.

ISSN 1740-2522 print/ISSN 1740-2530 online © 2005 Taylor & Francis Group Ltd
DOI: 10.1080/17402520500116723

each autoantibody separately. The SLE disease activity index (SLEDAI) is a clinical tool utilized to establish active disease based on clinical and laboratory findings in a recent period.

Flow-based, multiplex bead arrays (MBA) were recently developed from a variety of applications including the detection of ENA antibodies. This platform offers the potential for a rapid and sensitive method to assess multiple analytes in a single test. AtheNA multi-lyte test system is a homogeneous, multiplexed system for autoantibody analysis. It is a precise and easy to use technology for simultaneous quantative multi-autoantibody detection (Fulto et al. 1997). Polystyrene microparticles of uniform size are used as the solid phase. Beads are dyed using a defined combination of two dyes. The dying process results in 100 distinct bead sets. The intensity of the two colors identifies each bead set. Unique bead sets can be conjugated with various, unique target molecules of interest. Thousands of each bead set are combined to form a multiplex bead suspension. The bead suspension is added to the wells of a microplate. If the patient possesses an antibody to more than one bead set, all beads will be labeled with antibody and then the conjugate. The bead suspension is analyzed using the AtheNA Instrument.

Beads flow through the flow cell and light scatter determine discrete events. One laser identifies the amount of reporter on the surface. The other determines the amount of classification dye or identifies the bead set. Antigens included in the assay included: dsDNA, SSA/Ro, SSB/La, Sm, RNP, histone, centromere, Scl-70, and Jo-1.

The purpose of this study was to evaluate the AtheNA multi-lyte ANA system an MBA technology for the detection of ANA and ENA antibodies in the sera of SLE patients and healthy individuals. Clinically, the number and type of autoantibodies detected per patient was compared to the SLEDAI score for the presence of active disease.

We have recently shown the specificity of the multiplex system in Sjogren's syndrome (Gilburd et al. 2004), rheumatoid arthritis, scleroderma, and SLE (Shovman et al.). In this study, we chose to assess the clinical importance of an autoantibody profile in SLE patients. We confronted the question: Does an autoantibody profile aid in predicting a pattern of clinical manifestations and/or disease severity?

### Patients and methods

*Patients*

Sera samples from 199 consecutive and unselected individuals were tested utilizing the multiplex system. The sera samples were obtained from 113 patients with SLE and 86 healthy individuals obtained from the National Blood Bank.

*Definition of clinical features and disease activity of SLE patients*

All SLE patients fulfilled the ACR classification criteria for SLE (Tan et al. 1982). In 42 SLE patients, we compared the type, level and number of autoantibodies with clinical activity utilizing the SLEDAI score (Bombardier et al. 1992).

The patients attended the Lupus Clinic at our institution from 2000–2002. All patients underwent a documented medical interview and physical examination by a qualified rheumatologist. A serum sample from each patient was collected and the disease severity was assessed utilizing the SLEDAI score.

Clinical and serological data were obtained from the Lupus Clinic Database (Microsoft-Excel 2000).

*Autoantibody determination*

AtheNA Multi-Lyte ANA test System (Zeus Scientific, Inc. Raritan, NJ, USA) was utilized for simultaneous determination of autoantibodies to nine different antigens (dsDNA, SSA/Ro, SSB/La, Sm, RNP, Scl-70, Jo-1, centromere B, and histone).

The multiplex system is a diagnostic test which uses reagents that contain a mixture of different microsphere populations. Each of these populations are characterized by a specific red fluorescent color tone and a detection reagent on the surface. When incubated with a few microliters of a patient sample, each microsphere population captures its specific target molecule from the liquid phase. Analytes bound to the surface of the microspheres can be labeled using a green fluorescent labeling reagent, thus enabling the detection of many autoantibodies in one step.

Positive tests for all individual autoantibodies in the AtheNA Multi-Lyte ANA test System were defined as values greater than 120 units/ml (cutoff normal range).

*Statistical analysis*

Statistical analysis was performed using the STAT program. Descriptive statistics and linear correlation were utilized to compare between the various assays.

### Results

*Autoantibody profile*

*SLE sera*. Elevated titers of at least one autoantibody were found in 48% of SLE patients and elevated titers of anti-Ro antibodies were most commonly detected. The prevalence of specific autoantibodies was: anti-Ro- 23.8%, anti-dsDNA-19%, anti-histone- 19%, anti-RNP- 14.2%, anti-La antibodies- 11.9%, anti-Sm- 7.1%, anti-Scl 70-4.7%, and anti-centromere-2.4 (Table I). Utilizing ROC analysis, the sensitivity

Table I. Prevalence of the ENA profile utilizing the multiplex system*.

| Autoantibody | SLE sera (n-42) % | Literature* % |
|---|---|---|
| SSA | 23.8 | 21.4 |
| SSB | 11.9 | 7.1 |
| Sm | 7.1 | 10.7 |
| RNP | 14.2 | 32.1 |
| Histone | 19.0 | ND |
| Scl-70 | 4.7 | 0 |
| Centromere | 2.4 | ND |
| Jo-1 | 0.0 | ND |

*Martins et al. (2004).

and specificity of anti-DNA antibodies at a cutoff value = 34 IU/ml were 87.1% and 79.4% respectively. Elevated titers of anti-Jo1 antibody were not detected. Seven patients harbored one autoantibody only, 15 patients harbored 2–3 autoantibodies, 3 patients harbored 4–5 autoantibodies, and one patient harbored 6 autoantibodies.

*Normal sera*. The sera of 86 healthy individuals were tested by the AtheNA system. Elevated titers of anti-nuclear antibodies were detected in 3 (3%) sera from healthy subjects. None of the sera had anti-SS/A, anti-SS/B, anti-Jo-1, anti-dsDNA, anti-centromere or anti-histone activity. Anti-Sm, anti-RNP and anti-Scl-70 were detected each in one (1%) serum (not shown).

### *Autoantibody profile and clinical activity*

The autoantibody profile and clinical activity were evaluated in 42 SLE patients. Patients with an autoantibody load had more severe disease (Table II). Table III shows the distribution of autoantibodies in 26 SLE patients with elevated titers of at least one autoantibody.

A correlation was detected between the titer of anti-Ro antibodies and SLEDAI (Table IV). Patients with very high titers of anti-Ro antibodies also had more severe disease. We searched for the coupling of certain autoantibodies that may be found in elevated titers together. Four of five patients harbored both elevated titers of anti-Sm and anti-RNP antibodies. Elevated titers of anti-Ro and anti-La antibodies were found in 8 of 17 patients. The coupling of elevated titers of anti-RNP and anti-Sm antibodies was found in 4 of 6 patients. The number of autoantibodies in a single patient was not associated with specific manifestations of disease (nephritis, central nervous system involvement) but was associated with an elevated SLEDAI score.

Table II. Comparison of the presence of autoantibodies to the SLEDAI score in SLE patients.

| Number of patients | Number of AB | Mean SLEDAI score (range) |
|---|---|---|
| 22 | 1–3 | 5.1 (0–10) |
| 3 | 4–5 | 8 |
| 1 | 6 | 21 |

The patient with 6 different elevated autoantibodies and a high SLEDAI score is a 56 year old woman, married with 4 children, of a Yemenite descent. SLE was diagnosed 25 years ago, after her third delivery. Clinical manifestations of the disease include Jaccoud's arthropathy, discoid lupus, photosensitivity, livedo reticularis, digital ulcer, alopecia, pleural effusion, deep vein thrombosis, mild pancytopenia, sensorimotor poyneuropathy, low C3, C4, and elevated titers of anti-cardiolipin antibodies.

### Discussion

More than 100 autoantibodies are reported in the sera of SLE patients (Sherer et al. 2004). Some of those autoantibodies are pathogenic, have a role in the diagnosis of the disease and are utilized in the assessment of disease activity (Cervera and Shoenfeld). Although anti-dsDNA antibodies are the gold standard for SLE, some patients do not harbor elevated titers of anti-dsDNA antibodies and hence ENA detection is important. In our analysis of 9 autoantibodies by the multiplex system, elevated titers of at least one autoantibody were found in 48% of patients. Seven patients harbored one autoantibody only, 15 patients harbored 2–3 autoantibodies, 3 patients harbored 4–5 autoantibodies, and one patient harbored 6 autoantibodies. A correlation between the number of autoantibodies per patient and disease severity was found. The value of the presence of multiple autoantibodies in a single patient is not well established. One may suggest that the presence of many autoantibodies in a single patient could be associated with more severe disease. We found an association between the number of autoantibodies and the SLEDAI score. However, by utilizing the SLEDAI score alone, we were unable to detect a pattern of autantibodies associated with major organ involvement. Perhaps, clinical assessment at the onset of disease could be more informative.

The multiplex bead array system has recently been utilized for autoantibody detection in various autoimmune diseases (Martins et al. 2004). Thus, in our previous study, we compared the specificity and sensitivity of the recognition of ANA and 9 autoantibodies by the AtheNA system to the established ELISA method. Screening of samples obtained from healthy donors for ANA by AtheNA technology demonstrated a high concordance rate of 99% with the conventional ELISA test. Screening of disease related samples (systemic sclerosis, rheuma-

Table III. Comparison of autoantibody profile to the SLEDAI score in 26 SLE patients.

| SAMPLE | SSA | SSB | Sm | RNP | Scl 70 | dsDNA | Cent B | Histone | Nephritis | CNS | SLEDAI |
|---|---|---|---|---|---|---|---|---|---|---|---|
| 1 | + | | | | | + | | + | − | − | 11 |
| 2 | | | + | + | | | | + | − | − | 4 |
| 3 | + | | | | + | | | | − | − | 2 |
| 4 | + | + | | | | | | | − | − | 0 |
| 5 | + | | | | | | | | − | − | 13 |
| 6 | | | + | + | | + | | + | + | − | 8 |
| 7 | + | + | | | | | | | − | − | 10 |
| 8 | + | | | | | | | | − | + | 10 |
| 9 | + | | | + | | | | | − | − | 0 |
| 10 | + | + | | | | | | | − | − | 0 |
| 11 | + | | | | | | | | − | − | 10 |
| 12 | + | + | + | | | + | + | + | − | + | 21 |
| 13 | | | | | | + | | | + | − | 7 |
| 14 | | | + | + | + | + | | + | − | − | 6 |
| 15 | + | | | | | | | | − | − | 9 |
| 16 | + | + | | | | | | | − | − | 5 |
| 17 | | | | + | | + | | + | − | − | 8 |
| 18 | | | | | | + | | | − | − | 4 |
| 19 | + | + | | | | | | | − | − | 2 |
| 20 | + | | | | | | | + | − | − | 0 |
| 21 | | | | | | + | | + | − | + | 12 |
| 22 | | | | | | + | | + | − | − | 2 |
| 23 | | | + | + | | + | | + | + | − | 11 |
| 24 | + | + | | | | | | | + | − | 5 |
| 25 | + | | | | | | | | − | − | 10 |
| 26 | + | + | | | | | | | + | − | 11 |

toid arthritis) for ANA by technology also confirmed a high rate of concordance with the traditional ELISA (Shovman et al.). In another study, we assessed the multiplex system in 37 patients with Sjogren's syndrome. It was found to be sensitive and specific: Anti-Ro and anti-La antibodies were detected in 84% and 74% respectively, as described in the literature, while other autoantibodies were not found (Gilburd et al. 2004). In our current study, many autoantibodies were detected in patients indicating the diverse immune dysregulation in lupus as opposed to the prevalence of one single characteristic autoantibody encountered in organ specific autoimmune diseases such as Sjogren's syndrome. Interestingly, we found elevated titers of anti-Ro antibodies in 23.8% of patients that correlated with disease activity. Very elevated titers of anti-Ro antibodies may be useful in the assessment of disease activity especially in patients that do not present with elevated titers of anti-dsDNA autoantibodies.

Table IV. Anti-Ro antibody level correlates with SLEDAI score.

| Sample | SLEDAI score | Anti-Ro leve units/ml |
|---|---|---|
| 20 | 0 | 121 |
| 9 | 0 | 142 |
| 4 | 0 | 252 |
| 10 | 0 | 1362 |
| 24 | 1 | 205 |
| 3 | 2 | 132 |
| 19 | 2 | 258 |
| 16 | 5 | 1416 |
| 15 | 9 | 1087 |
| 8 | 10 | 224 |
| 11 | 10 | 262 |
| 25 | 10 | 699 |
| 7 | 10 | 1585 |
| 26 | 11 | 304 |
| 1 | 11 | 398 |
| 5 | 13 | 1323 |
| 12 | 21 | 1727 |

cut-off normal range < 120 units/ml.

In another recent study, with a multiplexed fluorescent microsphere immunoassay, reactivity to five of the most diagnostically useful ENA was measured in 249 serum samples, including samples from 56 patients previously documented to have SLE (Martins et al. 2004). Significant concentrations of 5 autoantibodies measured by the multiplexed assay in 56 SLE patients were similar to ours, except for RNP, where we had a lower frequency (Table I).

Routinely, autoantibodies specific for other autoimmune diseases, including anti-Jo1 antibodies for polymyositis, and anti-centromere and anti-Scl-70 antibodies for systemic sclerosis are not evaluated in SLE patients. Some studies imply a relation of these autoantibodies to clinical manifestations or disease activity in lupus patients (Al Attia and D'Souza 2003, Gussin et al. 2001). The importance of elevated titers of anti-Scl 70 and anti-centromere antibodies in SLE patients without overt clinical manifestations of another coexistent autoimmune disease is not known. Concomitantly, their presence could suggest an ensuing overlap syndrome.

Anti-Sm antibodies are rarely found without anti-RNP because both proteins associate with common snRNA species in the spliceosome. Anti-La is rarely detected without anti-Ro because both associate with a common type of RNA termed hYRNA (Egner 2000).

No autoantibody activity was detected in the majority of sera from normal individuals. This is in accordance to ANA that is normally found in 3–5% of the normal population and not indicative of ensuing autoimmune disease. Natural autoantibodies exist in low-medium titers, are of the IgM type, and hence bind with low affinity and avidity.

Considerable data confirms the advantage of the multiplexed technology and its applications in diverse fields of medicine including cancer research, cytokines, endocrinologic diseases, gene expression, genetic diseases, and infectious diseases (Feng et al. 2004, Rouquette et al. 2003. Keij and Steinkamp 1998, Earley et al. 2002, Dunbar 2003, Ye et al. 2001, Martins 2002, Pickering et al. 2002, 2002, de Jager 2003, Brailey and Susskind 2003, Chesterton et al. 2003, Pretl et al. 2003, Seideman and Peritt 2002). Our data suggests that the sensitivity and the precise technology of the AtheNA system makes it a feasible simultaneous detection of quantative multiple autoantibodies in a single micro titer, in the sera of SLE patients.

In conclusion, the multiplex system is specific and sensitive, provides an autoantibody profile in a single test, and may be useful as a diagnostic test for SLE. Clinical evaluation revealed that elevated anti-Ro antibodies are associated with severe disease and an autoantibody load may be indicative of more severe disease.

### References

Al Attia HM, D'Souza MS. 2003. Antitopoisomerase I antibody in patients with systemic lupus erythematosus/sicca syndrome without a concomitant scleroderma: Two case reports. Clin Rheumatol 22:70–72.

Bombardier C, Gladman DD, Urowitz MB, Caron D, Chang CH. 1992. And the committee on prognosis studies in SLE: Derivation of the SLEDAI. Arthritis Rheum 35:630–640.

Brailey P, Susskind BM. 2003. Accurate antibody specificity identification with single HLA antigen luminex beads. Hum Immunol 64:S117.

Cervera R, Shoenfeld Y, Pathogenic mechanisms. In: Peter J, Shoenfeld Y, editors. Autoantibodies. Amsterdam: Elsevier; 1996. p 607–617.

Chesterton KA, Pretl K, Sholander JT, Leffell MS, Zachary AA. 2003. Rapid and reliable detection of HLA-specific antibodies with the luminex platform. Hum Immunol 64:S108.

de Jager W, te Velthuis H, Prakken BJ, Kuis W, Rijkers GT. 2003. Simultaneous detection of 15 human cytokines in a single sample of stimulated peripheral bloodmononuclear cells. Clin Diagn Lab Immunol 10:133–139.

Dunbar SA, Vander Zee CA, Oliver KG, Karem KL, Jacobson JW. 2003. Quantitative, multiplexed detection of bacterial pathogens: DNA and protein applications of the Luminex LabMAP system. J Microbiol Methods 53:245–252.

Earley MC, Vogt RF Jr, Shapiro HM, Mandy FF, Kellar KL, Bellisario R, Pass KA, Marti GE, Stewart CC, Hannon WH. 2002. Report from a workshop on multianalyte microsphere assays. Cytometry 50:239–242.

Egner W. 2000. The use of laboratory tests in the diagnosis of SLE. J Clin Pathol 53:424–432.

Feng Y, Ke X, Ma R, Chen Y, Hu G, Liu F. 2004. Parallel detection of autoantibodies with microarrays in rheumatic diseases. Clin Chem 50:416–422.

Fulto RJ, McDade RL, Smith PL, Kienker LJ, Kettman JR. 1997. Advanced multiplexed analysis with the FlowMetrix TM system. Clin Chem 43:1749–1756.

Gilburd B, Abu-Shakra M, Shoenfeld Y, Giordano A, Bocci EB, delle Monache F, Gerli R. 2004. Autoantibodies profile in the sera of patients with Sjogren's syndrome: The ANA evaluation- a homogeneous, multiplexed system. Clin Dev Immunol 11:53–56.

Gussin HA, Ignat GP, Varga J, Teodorescu M. 2001. Anti-topoisomerase I (anti-Scl-70) antibodies in patients with systemic lupus erythematosus. Arthritis Rheum 44:376–383.

Keij JF, Steinkamp JA. 1998. Flow cytometric characterization and classification of multiple dual-color fluorescentmicrospheres using fluorescence lifetime. Cytometry 33:318–323.

Martins TB, Burlingame R, von Muhlen CA, Jaskowski TD, Litwin CM, Hill HR. 2004. Evaluation of multiplexed fluorescent microsphere immunoassay for detection of autoantibodies to nuclear antigens. Clin Diagn Lab Immunol 11:1054–1059.

Martins TB. 2002. Development of internal controls for the Luminex instrument as part of a multiplex seven-analyteviral respiratory antibody profile. Clin Diagn Lab Immunol 9:1–5.

Pickering JW, Martins TB, Greer RW, Schroder MC, Astill ME, Litwin CM, Hildreth SW, Hill HR. 2002. A multiplexed fluorescent microsphere immunoassay for antibodies to pneumococcal capsularpolysaccharides. Am J Clin Pathol 117:589–596.

Pickering JW, Martins TB, Schroder MC, Hill HR. 2002. Comparison of a multiplex flow cytometric assay with enzyme-linked immunosorbent assay for auantitation of antibodies to tetanus, diphtheria, and Haemophilus influenzae Type b. Clin Diagn Lab Immunol 9:872–876.

Pretl K, Chesterton KA, Sholander JT, Leffell MS, Zachary AA. 2003. Accurate, rapid charaterization of HLA-specific antibody using luminex technology. Hum Immunol 64:S108.

Rouquette AM, Desgruelles C, Laroche P. 2003. Evaluation of the new multiplexed immunoassay, FIDIS, for simultaneous quantitative determination of antinuclear antibodies and comparison with conventional methods. Am J Clin Pathol 120:676–681.

Seideman J, Peritt D. 2002. A novel monoclonal antibody screening method using the Luminex-100 microsphere system. J Immunol Methods 267:165–171.

Sherer Y, Gorstein A, Fritzler M, Shoenfeld Y. 2004. Autoantibody explosion in SLE: More than 100 different antibodies found in SLE patients. Semin Arthritis Rheum 34:501–537.

Shovman O, Gilburd B, Zandman-Goddard G, Yehiely A, Langevitz P, Shoenfeld Y. 2005. Multiplexed AtheNA Multi-Lyte immunoassay for ANA screening in autoimmune diseases. Autoimmunity 38:105–109.

Tan EM, Cohen AS, Fries JF, Masi AT, McShane DJ, Rothfield NF, Schaller JG, Talal N, Winchester RJ. 1982. The 1982 revised criteria for the classification of systemic lupus erythematosus. Arthritis Rheum 25:1271–1277.

Ye F, Li MS, Taylor JD, Nguyen Q, Colton HM, Casey WM, Wagner M, Weiner MP, Chen J. 2001. Fluorescent microsphere-based readout technology for multiplexed human single nucleotide polymorphism analysis and bacterial identification. Hum Mutat 17:305–316.

